# Proper use of noncontact infrared thermometry for temperature screening during COVID-19

**DOI:** 10.1038/s41598-021-90100-1

**Published:** 2021-06-04

**Authors:** Amber S. Hussain, Heather S. Hussain, Nathan Betcher, Robert Behm, Burt Cagir

**Affiliations:** 1 Department of General Surgery, Guthrie Robert Packer Hospital, 1 Guthrie Square, Sayre, PA 18840 USA; 2 Seattle, WA USA

**Keywords:** Disease prevention, Health policy

## Abstract

Among the myriad of challenges healthcare institutions face in dealing with coronavirus disease 2019 (COVID–19), screening for the detection of febrile persons entering facilities remains problematic, particularly when paired with CDC and WHO spatial distancing guidance. Aggressive source control measures during the outbreak of COVID-19 has led to re-purposed use of noncontact infrared thermometry (NCIT) for temperature screening. This study was commissioned to establish the efficacy of this technology for temperature screening by healthcare facilities. We conducted a prospective, observational, single-center study in a level II trauma center at the onset of the COVID-19 outbreak to assess (i) method agreement between NCIT and temporal artery reference temperature, (ii) diagnostic accuracy of NCIT in detecting referent temperature $\ge 100.0\,^{\circ }{\mathrm{F}}$ and ensuing test sensitivity and specificity and (iii) technical limitations of this technology. Of 51 healthy, non-febrile, healthcare workers surveyed, the mean temporal artery temperature was $98.4\,^{\circ }{\mathrm{F}}$ ($95\%$ confidence interval (CI) = $[98.2,98.6]\,^{\circ }{\mathrm{F}}$). Mean NCIT temperatures measured from ${1}\,{\mathrm{ft}}$, ${3}\,{\mathrm{ft}}$, and ${6}\,{\mathrm{ft}}$ distances were $92.2\,^{\circ }{\mathrm{F}}$ $(95\%\ {\text {CI}}=[91.8\ 92.67]\,^{\circ }{\mathrm{F}})$, $91.3\,^{\circ }{\mathrm{F}}$ $(95\%\ {\text {CI}}=[90.8\ 91.8]\,^{\circ }{\mathrm{F}})$, and $89.6\,^{\circ }{\mathrm{F}}$ $(95\%\ {\text {CI}}=[89.2 \ 90.1]\,^{\circ }{\mathrm{F}})$, respectively. From statistical analysis, the only method in sufficient agreement with the reference standard was NCIT at ${1}\,{\mathrm{ft}}$. This demonstrated that the device offset (mean temperature difference) between these methods was $-6.15\,^{\circ }{\mathrm{F}}$ ($95\%\ {\text {CI}}=[-6.56,-5.74]\,^{\circ }{\mathrm{F}}$) with 95% of measurement differences within $-8.99\,^{\circ }{\mathrm{F}}$ ($95\%\ {\text {CI}}=[-9.69,-8.29]\,^{\circ }{\mathrm{F}}$) and $-3.31\,^{\circ }{\mathrm{F}}$ ($95\%\ {\text {CI}}= [-4.00,-2.61]\,^{\circ }{\mathrm{F}}$). By setting the NCIT screening threshold to $93.5\,^{\circ }{\mathrm{F}}$ at ${1}\,{\mathrm{ft}}$, we achieve diagnostic accuracy with $70.9\%$ test sensitivity and specificity for temperature detection $\ge 100.0\,^{\circ }{\mathrm{F}}$ by reference standard. In comparison, reducing this screening criterion to the lower limit of the device-specific offset, such as $91.1\,^{\circ }{\mathrm{F}}$, produces a highly sensitive screening test at $98.2\%$, which may be favorable in high-risk pandemic disease. For future consideration, an infrared device with a higher distance-to-spot size ratio approaching 50:1 would theoretically produce similar results at ${6}\,{\mathrm{ft}}$, in accordance with CDC and WHO spatial distancing guidelines.

## Introduction

Since the emergence of COVID-19, over 100 guidance documents have been produced by the World Health Organization (WHO) and the Centers for Disease Control and Prevention (CDC)^1,2^. Interim guidance for United States healthcare facilities recommend aggressive universal source control measures and well-equipped triage procedures at the entrances of facilities to actively screen individuals for fever. Fever is defined as either measured temperature greater than or equal to $100.0\,^{\circ }{\mathrm{F}}$ or subjective fever^1^ and symptoms. Respiratory symptoms consistent with COVID-19 are cough, shortness of breath, and sore throat^1^. Maintaining spatial separation at ${6}\,{\mathrm{ft}}$ is also advised by the CDC and keeping at least ${1}\, {\mathrm{m}}$ apart is recommended by the WHO^1–3^.

Under this guidance, our center aimed to investigate the diagnostic accuracy of temperature measurement using the Fluke 561 Noncontact Infrared Thermometer (NCIT) from a distance of ${6}\,{\mathrm{ft}}$. A cursory investigation into this device’s use revealed variable results in temperature measurement, which degraded as a function of distance, and a notable offset compared to the expected body temperature.

Furthermore, there is a paucity of high-quality research comparing thermal measurements obtained by noncontact infrared thermometry (IRT) versus conventional methods in public health applications, such as pandemic disease, like COVID-19.

Several studies support this dissimilitude in diagnostic accuracy across a myriad of available devices^4–6^. In a review of six studies by Bitar et al.^7^ assessing measurement of forehead temperature using an NCIT, test sensitivity ranged widely from 4 to 89.6%, the specificity from 75.4 to 99.6%, and the Positive Predictive Value (PPV) between 3.5 and $65.4\%$. Four studies failed to provide technical information about the NCIT device used. Three studies failed to report environmental conditions or stabilization factors, and a majority of studies did not detail the procedural methods employed for measurement testing, such as distance away from the target. Studies using handheld devices at less than ${7.9}\,{\mathrm{in}}$ (${20}\,{\mathrm{cm}}$) from the forehead showed improved accuracy^4,8^.

This variability called into question the efficacy of screening practices that similarly equipped healthcare facilities are implementing to reduce the spread of COVID-19 and comply with spatial distancing guidance. This study will analyze temperature data collected using the Fluke 561 infrared thermometer at ${1}\,{\mathrm{ft}}$, ${3}\,{\mathrm{ft}}$ and ${6}\,{\mathrm{ft}}$ distances to assess the accuracy of this device compared to conventional temporal artery thermometry and survey the technological constraints inherent to NCITs. Our intent is to inform a concerned audience of explicit and implicit limitations and recommend an optimal screening process for infection prevention and control measures during pandemic disease.

## Methods

### Study design

This nonblinded prospective single-center study was designed to examine the validity of the Fluke 561 NCIT (Fluke Corp, Everett, WA) at variable distances compared to conventional temporal artery reference temperature of employees in a U.S. healthcare facility during the outbreak of COVID-19.

The study was conducted at Guthrie Robert Packer Hospital, a rural 267-bed tertiary care level II trauma center serving the southern tier of New York and the northern tier of Pennsylvania^9^.

Participants were recruited by study investigators at random from different hospital wards. An institutional review board-approved letter was used to inform participants about the study and informed consent was obtained prior to enrollment.

#### Ethical approval

The study protocol and informed consent documentation were reviewed and approved by the Institutional Review Board of The Guthrie Clinic. All procedures followed were in accordance with the ethical standards of the responsible committee on human experimentation and with the Helsinki Declaration of 1964 and its later amendments.

#### Participants

Eligible participants were healthy adults aged 18 or older who were employed by the Guthrie Clinic, working on-site during the COVID-19 pandemic. Participants were excluded if they were pregnant.

### Procedures and data collection

Data was collected, by a single researcher, between April 6 and April 10, 2020 on different hospital wards. Age and gender were included in data collection.

A fever threshold $\ge 100.0\,^{\circ }{\mathrm{F}}$ was adopted in accordance with CDC guidelines for U.S. healthcare facility screening^1^. If participants were found to have a measured temperature $\ge 100.0\,^{\circ }{\mathrm{F}}$, they would be instructed to report to the Employee Health Office.

Mode of use, device calibration, temperature scanning and disinfection were followed according to manufacturer instructions for each apparatus and in compliance with CDC infection prevention standards. Similarly, Personal Protective Equipment (PPE) compliant with institution and CDC precautions for COVID-19, including the use of an N95 respirator, was worn.

#### Temporal artery thermometer (TAT)

Each participants’ baseline body temperature was measured using the Exergen TemporalScanner (Model TAT5000, Exergen Corp, Watertown, MA) Temporal Artery Thermometer (TAT). Technical specifications for the Exergen TemporalScanner are given in Table 1.

**Table 1:** Technical specifications of temporal artery and non-contact infrared thermometers.

Device	Exergen TemporalScanner	Fluke 561 (NCIT)	Fluke 568 (NCIT)$^{\mathrm{a}}$
Temperature range	$[61,110]\,^{\circ }{\mathrm{F}}$ $([16,43]\,^{\circ }\hbox {C})$	$[-40,1022]\,^{\circ }{\mathrm{F}}$ ($[-40,550]\,^{\circ }\hbox {C}$)	$[-40,1472]\,^{\circ }{\mathrm{F}}$ ($[-40,800]\,^{\circ }\hbox {C}$)
Accuracy	$\pm 0.2\,^{\circ }{\mathrm{F}}$ or $0.1\,^{\circ }\hbox {C}^{\mathrm{b}}$	$>32\,^{\circ }{\mathrm{F}}: \max (\pm 1\%,\pm 2\,^{\circ }{\mathrm{F}})$	$>32\,^{\circ }{\mathrm{F}}: \max (\pm 1\%,\pm 2\,^{\circ }{\mathrm{F}})$
Repeatability	Not available	$\max (\pm 0.5\%,\pm 2\,^{\circ }{\mathrm{F}} \ (1\,^{\circ }\hbox {C})$	$\max (\pm 0.5\%,\pm 1\,^{\circ }{\mathrm{F}} \ (0.5\,^{\circ }\hbox {C}))$
${\text {D:S}}\ (90\%)$	Not applicable	12:1	50:1
Response time	$40 \ {\mathrm{ms}}$	$<500 \ {\mathrm{ms}}$	$<500 \ {\mathrm{ms}}$
Emissivity	Not applicable	Lo (0.3)/Med (0.7)/Hi (0.95)	0.10 to 1.00 by 0.01
Resolution	$0.1\,^{\circ }{\mathrm{F}}$ or $^{\circ }\hbox {C}$	$0.1\,^{\circ }{\mathrm{F}}$ or $^{\circ }{\mathrm{C}}$	$0.1\,^{\circ }{\mathrm{F}}$ or $^{\circ }\hbox {C}$

$^{\mathrm{a}}$For reference and discussion only, device was not tested in this study.

$$^{\mathrm{b}}$$Per ASTM E1112.

This accurate and noninvasive thermometer uses infrared technology to measure heat emitted from the skin’s surface overlying the temporal artery^10^. Temporal artery temperature is a close approximate of rectal temperature and therefore accurately reflects a measure of core body temperature^10–12^.

#### Non-contact infrared thermometer (NCIT)

Next, a digital ruler was used to measure and mark ${1}\,{\mathrm{ft}}$, ${3}\,{\mathrm{ft}}$, and ${6}\,{\mathrm{ft}}$ distances on the floor of each testing location, relative to a fixed reference point.

**Figure 1:**
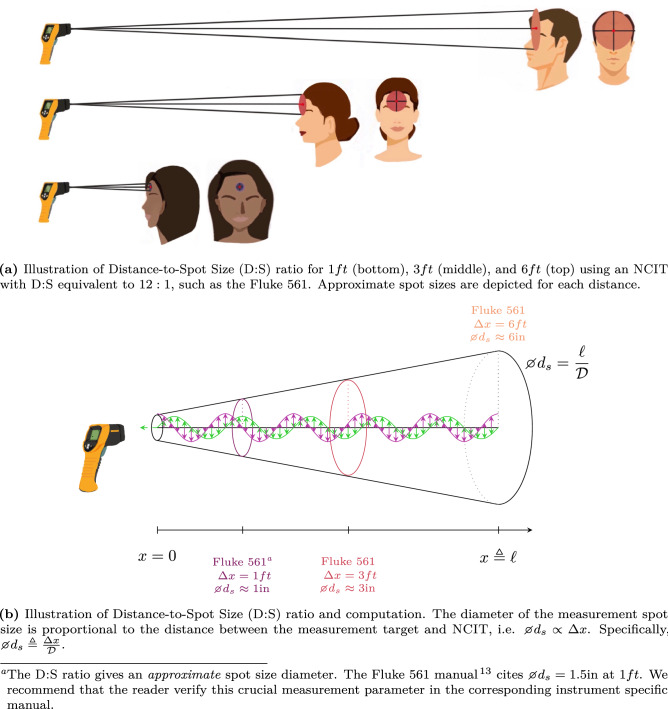
Illustration of distance-to-spot size (D:S) ratio.

Three sequential measurements were obtained using the Fluke 561 NCIT at device-to-target [Target, in this context, denotes the forehead of each study participant at the specified IR measurement distance] distances of ${6}\,{\mathrm{ft}}$, ${3}\,{\mathrm{ft}}$, and ${1}\,{\mathrm{ft}}$ (see Fig. 1a) with emissivity set to Hi (see Table 1). The focal point of the device, marked by a laser, was centered on each participants’ forehead whom were asked to close their eyes during collection.

NCITs measure the amount of infrared energy emitted by an object’s surface; it is not a direct measure of core body temperature and can be influenced by environmental conditions and physiological factors.

To ensure accurate temperature measurement, device-to-target distance is crucial since it governs the measurement spot size according to the NCIT’s distance-to-spot size (D:S) specification. The spot size, i.e. diameter of the measurement area, indicates 90% encircled energy^13^ and increases proportionally with distance. For that reason, the spot size should not exceed the intended measurement area [Fluke recommends that the measurement area be twice as large as the measured spot size^14^]. The Fluke 561 D:S ratio is 12:1 which implies a ${6}\, {\mathrm{in}}$ spot diameter at ${6}\,{\mathrm{ft}}$ (see Fig. 1)^13^.

#### Outcome measures

Primary outcome measures were temperature values obtained with the Exergen TAT [For brevity, TAT and NCIT may be used interchangeably in reference to the Exergen TemporalScanner and Fluke 561 devices, respectively] and three temperature measurements with the Fluke 561 at ${1}\,{\mathrm{ft}}$, ${3}\,{\mathrm{ft}}$, and ${6}\,{\mathrm{ft}}$ distances. NCIT temperatures were compared to temporal artery reference temperatures and analyses of agreement were conducted. Secondary outcome measures of test sensitivity and specificity to assess diagnostic accuracy for detecting temperature $\ge 100.0\,^{\circ }{\mathrm{F}}$ by TAT were then analyzed using NCIT screening criteria on an open source data set. Third, this paper outlines the salient technical features of noncontact infrared thermometry.

#### Sample size

The sample size for this study (N = 51) exceeded minimum requirements for all statistical tests and the Bland–Altman agreement analyses. The degree of freedom for this study was 50 which corresponds to a *t*-value of 2.009 used in Confidence Interval (95% CI) computations.

#### Blinding

All participants and data collectors were unblinded to data collection. Fully deidentified objective outcome data was provided to an independent data analyst.

### Data analysis

All data analyses were performed using MATLAB, version R2019a (The MathWorks Inc., Natick, MA). First, the validity of NCIT data at ${1}\,{\mathrm{ft}}$, ${3}\,{\mathrm{ft}}$, and ${6}\,{\mathrm{ft}}$ had to be established. Central tendency and dispersion characteristics for each dataset was computed and graphically visualized through histogram plots. Formal judgment of normality was performed using robust statistical testing. Those results, in combination with the limitations of the NCIT device, led to rejection of NCIT measurements at ${3}\,{\mathrm{ft}}$ and ${6}\,{\mathrm{ft}}$. Then, methodological comparison between TAT and NCIT at 1 ft was conducted using Bland–Altman plots, the benchmark approach for assessing between-method differences^15–17^. This provided an indication of bias and 95% Limits of Agreement (LOA) between the measurement methods. Diagnostic accuracy for detecting fever was then explored.

Prior to presenting the main results, we introduce the following nomenclature: $T_{\mathcal {X}} \,(^{\circ }{\mathrm{F}})$ denotes the temperature measured by method ${\mathcal {X}}$. That is, $T_{\text {TAT}}$ represents temperature measured by the TAT, while $T_{1\,{\mathrm{ft}}}$, $T_{3\,{\mathrm{ft}}}$, and $T_{6\,{\mathrm{ft}}}$ correspond to NCIT measurements.

## Results

A total of 51 healthy adults were sampled. Ages ranged 21 to 67 with a median age of 31[Median excludes one participant who did not report their age.]. Of the participants, 37 $(72.5\%)$ were female and none exhibited fever or symptoms of illness ($N=51$, $P = \varnothing$). The mean temporal artery temperature was $98.4\,^{\circ }{\mathrm{F}}$ with all measurements falling between 97.1 and $99.6\,^{\circ }{\mathrm{F}}$. No data was excluded from the study.

**Table 2:** Descriptive statistics for each measurement group.

Measurement method	Mean ($^{\circ }{\mathrm{F}}$)	SD$^{\mathrm{a}}$ ($^{\circ }{\mathrm{F}}$)	Median ($^{\circ }{\mathrm{F}}$)	Range ($^{\circ }{\mathrm{F}}$)	IQR ($^{\circ }{\mathrm{F}})^{\mathrm{b}}$	Min($^{\circ }{\mathrm{F}}$)	Max ($^{\circ }{\mathrm{F}}$)
*Temporal* (TAT)	98.4	0.59	98.4	2.5	0.6	97.1	99.6
$\Delta x = 1\,{\mathrm{ft}}$ (NCIT)	92.2	1.52	92.4	6.4	2.5	88.9	95.3
$\Delta x = 3\,{\mathrm{ft}}$ (NCIT)	91.3	1.72	91.8	6.8	2.8	87.4	94.2
$\Delta x = 6\,{\mathrm{ft}}$ (NCIT)	89.6	1.68	89.9	6.3	2.9	86.6	92.9

$^{\mathrm{a}}$ *SD*: Standard Deviation.

$^{\mathrm{b}}$ *IQR*: Interquartile Range.

### Descriptive statistics

**Figure 2:**
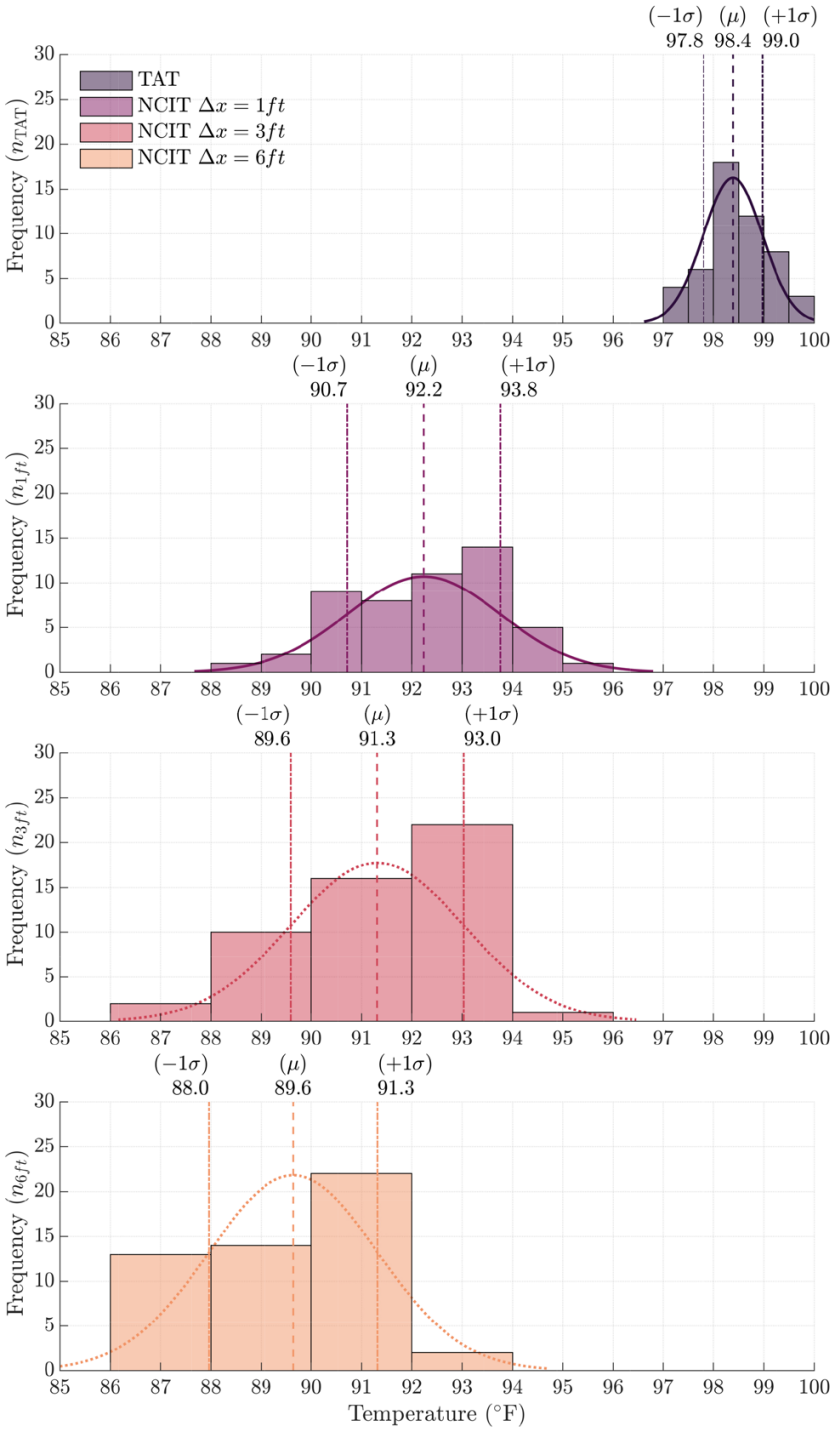
Overlay of histograms and normal density function fits for each measurement method. Vertical dashed lines annotate the mean of each measurement method and vertical dotted lines represent one standard deviation.

Measures of central tendency and dispersion characteristics for each measurement method were computed and summarized in Table 2. Histograms for each measurement method are provided in Fig. 2 where $T_{\mathcal {X}} (^{\circ }{\mathrm{F}})$ was binned with width defined by $w_{bin}=\frac{3.49 \sigma }{\root 3 \of {n}}$.

This figure illustrates the existence of a measurement bias, i.e. shift in the mean, between TAT and NCIT measurements. Mean temperature for the TAT was $98.4\,^{\circ }{\mathrm{F}}$ $(95\%\ {\text {CI}}=[98.2\ 98.6]\,^{\circ }{\mathrm{F}})$. The mean temperatures for NCIT at ${1}\, {\mathrm{ft}}$, ${3}\,{\mathrm{ft}}$, and ${6}\,{\mathrm{ft}}$ were $92.2\,^{\circ }{\mathrm{F}}$ $(95\%\ {\text {CI}}=[91.8\ 92.7]\,^{\circ }{\mathrm{F}})$, $91.3\,^{\circ }{\mathrm{F}}$ $(95\%\ {\text {CI}}=[90.8\ 91.8]\,^{\circ }{\mathrm{F}})$, and $89.6\,^{\circ }{\mathrm{F}}$ $(95\%\ {\text {CI}}=[89.2 \ 90.1]\,^{\circ }{\mathrm{F}})$, respectively.

The dispersion of NCIT measurements is greater compared to the central TAT method. Specifically, the standard deviation ($\sigma$) for TAT is 0.6 ($95\%\ {\text {CI}} = [0.5, 0.7]$) compared to the NCIT where $\sigma = 1.5$ ($95\%\ {\text {CI}} = [1.3,1.9]$) at 1 ft, and $\sigma = 1.7$ ($95\%\ {\text {CI}} = [1.4, 2.1]$) for both the ${3}\,{\mathrm{ft}}$ and ${6}\,{\mathrm{ft}}$ measurements.

No distributions displayed readily apparent asymmetry or skew; however, formal statistical tests are performed in the next section to determine whether TAT and NCIT measurements are drawn from a normally distributed population.

### Distribution tests for normality

Statistical test decisions, along with corresponding *p* values, are summarized in Table 3 for the Lilliefors’ Composite and Anderson–Darling Goodness of Fit (AD GOF) hypothesis tests.

**Table 3:** Distribution tests of each measurement group.

Measurement method	Lilliefors test	Anderson–Darling GOF
*Temporal* (TAT)	False ($p = 0.19$)	False ($p = 0.28$)
$\Delta x = 1$ ft (NCIT)	False ($p = 0.25$)	False ($p = 0.11$)
$\Delta x = 3$ ft (NCIT)	True ($p = 0.03$)	True ($p = 0.008$)
$\Delta x = 6$ ft (NCIT)	True ($p = 0.04$)	True ($p = 0.005$)

Lilliefors and AD GOF test outcomes for both TAT ($p=0.19$ and $p=0.28$) and NCIT at ${1}\,{\mathrm{ft}}$ ($p=0.25$ and $p=0.11$) were false, indicating that the null hypothesis, data comes from a normal distribution, cannot be rejected at the $5\%$ significance level. Conversely, the Lillefors and AD GOF results for NCIT measurements at ${3}\,{\mathrm{ft}}$ and ${6}\,{\mathrm{ft}}$ ($p<0.1$, $p<0.05$) reject the same null hypothesis at the $5\%$ significance level.

Thus, ${1}\,{\mathrm{ft}}$ NCIT data is the only measurement set whose distribution coincides with the referent method’s distribution.

Non-normal distributions of ${3}\,{\mathrm{ft}}$ and ${6}\,{\mathrm{ft}}$ NCIT data combined with insufficient distance-to-spot size ratings [The corresponding spot sizes were approximately 3 in and 6 in, respectively, which exceed reliable forehead area thresholds] for the intended measurement target using the Fluke 561 (see Fig. 1 and “Basics of noncontact infrared thermal measurements” section), invalidate the ${3} \,{\mathrm{ft}}$ and ${6}\,{\mathrm{ft}}$ NCIT procedures for temperature screening or further method comparison.

### Bland–Altman method comparison

**Figure 3:**
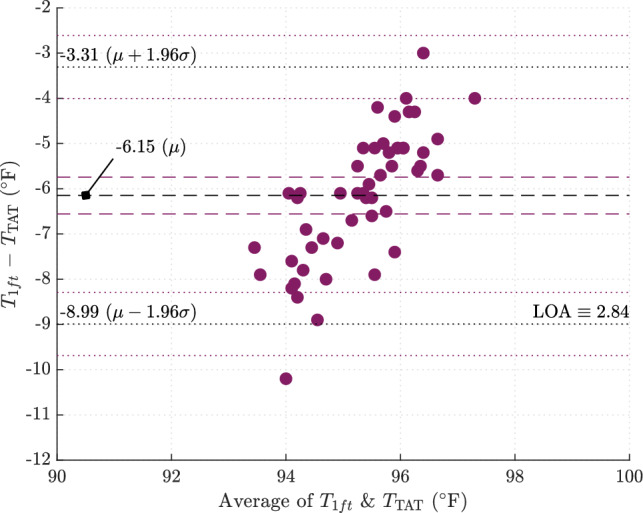
Bland–Altman method comparison plot for IR measurement at $1\,{\mathrm{ft}}$ versus temporal measurement.

A Bland–Altman plot is provided in Fig. 3 for the ${1}\,{\mathrm{ft}}$ NCIT measurement in comparison with the reference (TAT) method. The difference between the IR measurement and temporal measurement is plotted as the dependent variable, with the average of the two measurements serving as the independent variable. The mean bias (dashed line) and $95\%$ LOA (dotted lines) are illustrated with corresponding 95% CIs.

The mean difference between NCIT at ${1}\,{\mathrm{ft}}$ and TAT is $-6.15\,^{\circ }{\mathrm{F}}\ (95\%\ {\text {CI}}= [-6.56, -5.74]\,^{\circ }{\mathrm{F}})$ with LOA equivalent to $2.84\,^{\circ }{\mathrm{F}}$ $(95\%\ {\text {CI}}= [2.14, 3.54]\,^{\circ }{\mathrm{F}})$. In other words, $95\%$ of the measurement differences at ${1}\,{\mathrm{ft}}$ fall within $-8.99\,^{\circ }{\mathrm{F}}$ $(95\%\ {\text {CI}}= [-9.69,-8.29]\, ^{\circ }{\mathrm{F}})$ and $-3.31\,^{\circ }{\mathrm{F}}$ $(95\%\ {\text {CI}}= [-4.00,-2.61] \,^{\circ }{\mathrm{F}})$ of the TAT measurement.

### Assessing diagnostic accuracy

In this section, we quantify test sensitivity and specificity of NCIT at ${1}\,{\mathrm{ft}}$ for detecting temperature $\ge 100.0\,^{\circ }{\mathrm{F}}$ by the reference method based on NCIT screening thresholds.

**Figure 4:**
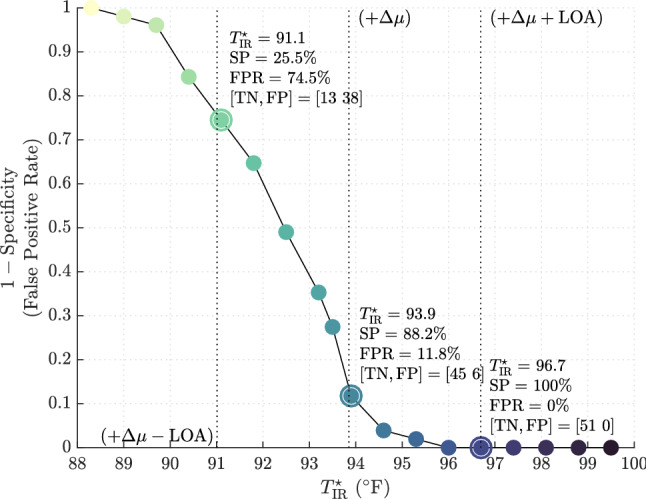
Specificity of fever screening protocol for varying NCIT temperature thresholds in determining afebrile observations.

With a mean measurement bias ($-6.15\,^{\circ }{\mathrm{F}}$) and LOA ($2.84\,^{\circ }{\mathrm{F}}$) established using Bland–Altman, diagnostic specificity can be computed for various NCIT thresholds, denoted $T^\star _{\text {IR}}$. Figure 4 demonstrates the effect $T^\star _{\text {IR}}$ has on specificity (*SP*) with threshold values encircled and presented in Table 4. For example, $T^\star _{\text {IR}}= 93.9\,^{\circ }{\mathrm{F}}$ corresponds to a test specificity of $88.2\%$ (FPR = $11.8\%$) with True Negative (TN) and False Positive (FP) group sizes equal to 45 and 6, respectively.

**Table 4:** Diagnostic accuracy for detecting referent temperature above $100.0\,^{\circ }{\mathrm{F}}$ using NCIT screening thresholds ($T^\star _{\text {IR}}$) of $91.1\,^{\circ }{\mathrm{F}}$ (left), $93.9\,^{\circ }{\mathrm{F}}$ (middle), and $96.7\,^{\circ }{\mathrm{F}}$ (right). These represent the mean, and upper and lower limits of agreement.

	$T^\star _{\text {IR}}=91.1\,^{\circ }{\mathrm{F}}$	$T^\star _{\text {IR}}=93.9\,^{\circ }{\mathrm{F}}$	$T^\star _{\text {IR}}=96.7\,^{\circ }{\mathrm{F}}$
**Specificity of NCIT at 1 ft**^**a**^			
SP$^{\mathrm{b}}$ (%)	25.5	88.2	100
FPR (%)	74.5	11.8	0
TN (*n*)	13	45	51
FP (*n*)	38	6	0

$$^{\mathrm{a}}T^\star _{\text {IR}}\triangleq T^\star +\Delta \mu + \delta T,\ \Delta \mu = -6.15 \,^{\circ }{\mathrm{F}},\ T^\star = 100.0\,^{\circ }{\mathrm{F}}$$.

$^{\mathrm{b}}$ Specificity (SP), False Positive Rate (FPR), True Negative (TN), False Positive (FP).

#### Open source data analysis—STRIDE cohort

Given the sampled population was constrained to a group of healthy adults, complete assessment of diagnostic accuracy, including test sensitivity, necessitates a population with fever occurrence. Thus, an open source cohort from the Stanford Translational Research Integrated Database Environment (STRIDE)^18^ was imported and synthesized to validate this study’s findings on a larger population with fever prevalence. The STRIDE database is comprised of 578,522 adult outpatient temperature measurements at Stanford Health Care, gathered between 2007 and 2017. The mean of the STRIDE Cohort was $98.0\,^{\circ }{\mathrm{F}}$ with a standard deviation of $0.7\,^{\circ }{\mathrm{F}}$.

The method of measurement used in the STRIDE cohort was oral thermometry. Therefore, the cohort observations were adjusted by $+0.8\,^{\circ }{\mathrm{F}}$ to account for differences between oral and Exergen TAT thermometry^10,12^. The adjusted mean is $98.8\,^{\circ }{\mathrm{F}}$ with $P = 19{,}584$ febrile observations.

A simulated ${1}\,{\mathrm{ft}}$ NCIT measurement set is then synthesized with the adjusted cohort and repeated random sampling from the distribution

1 $$\begin{aligned} \phi (\Delta T) = \tfrac{\rho }{ \sqrt{2\pi }}{\text {e}}^{-\frac{1}{2} \left( \frac{\Delta T-\Delta \mu }{\rho }\right) ^2} \end{aligned}$$

where $\Delta \mu \triangleq -6.15\,^{\circ }{\mathrm{F}}$ and $\rho \triangleq \tfrac{{LOA}}{1.96}= 1.45\,^{\circ }{\mathrm{F}}$. Conceptually, this is a transformation producing NCIT measurements for the open source cohort.

Diagnostic accuracy for detecting referent temperature greater than $100.0\,^{\circ }{\mathrm{F}}$ using three different NCIT temperature thresholds is summarized in Table 5.

**Table 5:** Diagnostic accuracy for detecting referent temperature above $100.0\,^{\circ }{\mathrm{F}}$ using different NCIT screening thresholds ($T^\star _{\text {IR}}$) of $91.1\,^{\circ }{\mathrm{F}}$ (left), $93.9\,^{\circ }{\mathrm{F}}$ (middle), and $96.7\,^{\circ }{\mathrm{F}}$ (right).

	$T^\star _{\text {IR}}=91.1\,^{\circ }{\mathrm{F}}$	$T^\star _{\text {IR}}=93.9\,^{\circ }{\mathrm{F}}$	$T^\star _{\text {IR}}=96.7\,^{\circ }{\mathrm{F}}$
**Diagnostic accuracy of NCIT at 1 ft**^**a**^ **(Synthesized STRIDE cohort)**			
SP$^{\mathrm{b}}$ (%)	17.1	80	99.6
SE (%)	98.2	60.1	6.56
FPR (%)	82.9	20	0.39
FNR (%)	1.76	39.9	93.4
PPV (%)	3.99	9.54	37.1
NPV (%)	99.6	98.3	96.8
TN (*n*)	95, 620	446, 976	556, 458
FP (*n*)	463,017	111,661	2179
FN (*n*)	343	7809	18, 230
TP (*n*)	19, 241	11, 776	1285

$$^{\mathrm{a}}T^\star _{\text {IR}}\triangleq T^\star +\Delta \mu + \delta T,\ \Delta \mu = -6.15\,^{\circ }{\mathrm{F}},\ T^\star = 100.0\,^{\circ }{\mathrm{F}}$$.

$^{\mathrm{b}}$ Specificity (SP), Sensitivity (SE), False Positive Rate (FPR), False Negative Rate (FNR), Positive Predictive Value (PPV), Negative Predictive Value (NPV), True Negative (TN), False Positive (FP), False Negative (FN), True Positive (TP).

**Figure 5:**
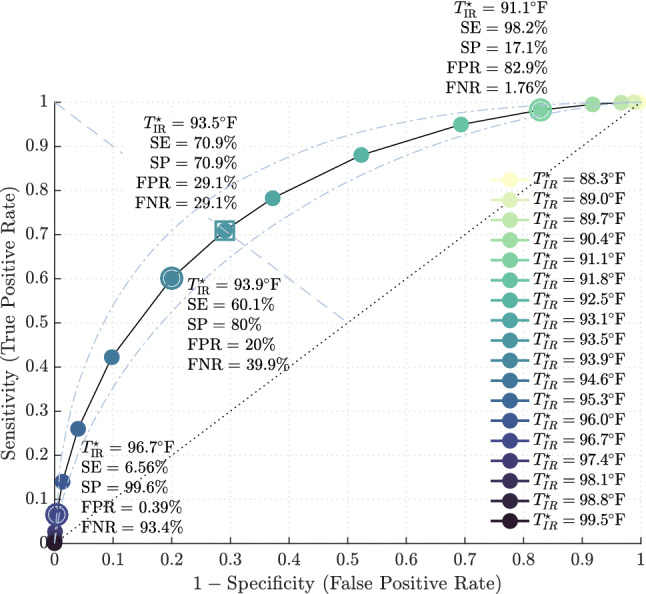
ROC curve for detecting temperature $\ge 100.0\,^{\circ }{\mathrm{F}}$ for fever screening using open source cohort^18^ based on NCIT threshold value. Sensitivity and specificity for points $91.1\,^{\circ }{\mathrm{F}}$ (right), $93.9\,^{\circ }{\mathrm{F}}$ (middle), and $96.7\,^{\circ }{\mathrm{F}}$ (left) are encircled. The dashed line represents a non-discriminatory test.

A receiver operating characteristic (ROC) curve is shown in Fig. 5 with the points in Table 5 encircled. This graph plots test sensitivity over (1-specificity). This provides a graphical depiction of the implicit trade-off between sensitivity and specificity at select screening criteria.

For the Fluke 561, this curve provides a visual guide for apriori selection of fever screening criteria adjusted to an acceptable sensitivity and specificity for a certain clinical context.

## Discussion

### Basics of noncontact infrared thermal measurements

*Thermal radiation (first principles)* An infrared thermometer measures the thermal radiation energy of an object and computes the temperature according to the fundamental Stefan–Boltzman law

2 $$\begin{aligned} M = \varepsilon \sigma {\mathbb {T}}^4 \end{aligned}$$

where *M* is the radiant exitance, a measure of radiation power emitted by an object into an imperfect vacuum, $\sigma = 5.670373\times 10^{-1}\, {\mathrm{W}}\,{\mathrm{m}}^{-2}\,{\mathrm{K}}^{-4}$, $\varepsilon$ denotes the emissivity of the emitting object, and ${\mathbb {T}}\,({\mathrm{K}})$ represents its absolute Temperature^19^. The emissivity $\varepsilon$ is a measure of how well an object can emit energy as thermal radiation. Almost perfect emitters, such as skin, have high emissivity ($\varepsilon _{\text {skin}} = 0.98$) while highly reflective surfaces such as polished metals are low.

Sources of error in the computation shown in Eq. (2) stem from the focal resolution of the device, introduced in “Non-contact infrared thermometer (NCIT)” section and discussed further below, along with the prescribed $\varepsilon$ value which can lead to large temperature errors due to the fourth power dependence.

Choosing a device with a sufficient D:S ratio such that the spot size is completely inscribed by the measurement area is crucial. If the spot size exceeds the target measurement area, such as the participant’s forehead, then the NCIT samples extraneous thermal radiation leading to incorrect forehead temperature measurements.

Lastly, thermal radiation is subject to atmospheric interference and distortion. End users should mitigate the effects of environmental factors affecting measurement error^5^, such as controlling for ambient temperature gradients, minimizing surface irregularity, and stabilizing device-to-target alignment in an effort to prevent scattering.

### Discussion of results

Infected patients are the primary source of pathogen dissemination in healthcare settings^3^. If initial screening and containment efforts fail, the ramifications can be particularly severe. Healthcare personnel may fall ill and transmit disease to others, including high-risk patients^2^. Infected staff may also require healthcare services themselves, placing additional strain on the medical system as healthcare providers become healthcare receivers.

In a case series by Wang et al.^20^ of 138 hospitalized patients with COVID-19, 41% were presumed to be due to hospital-related transmission, affecting 40 (29%) health professionals and 17 $(12.3\%)$ hospitalized patients.

The lack of a highly sensitive screening test for COVID-19 undermines efforts to contain viral spread^21^, an issue compounded by imperfect COVID-19 diagnostic tests with low-moderate sensitivities estimated around 70%^22^. In a study analyzing 1099 COVID-19 patients in China this year, fever was present in $43.8\%$ of cases upon admission and $88.7\%$ during hospitalization^23^.

A highly sensitive screening strategy allows timely detection of outbreaks and detects almost all cases of pandemic illness, which thereby limits disease spread and optimizes infection prevention and control measures. This approach, however, is much more resource and time consuming as a consequently higher rate of false positive tests require second or multi-stage screening surveys or confirmatory diagnostic testing to correctly classify diseased versus nondiseased individuals.

Alternatively, employing a screening strategy with lower sensitivity (i.e., lower level of detection) affords higher feasibility but at the cost of a higher false negative test rate^24^. Using this approach, a negative test result cannot safely assume someone is uninfected^22^. This surveillance method is discouraged and more consequential since misdiagnosed infected persons are not identified or contained by screening strategies and can go on to infect others^22^.

Results in this study show that utilizing the Fluke 561 within manufacturer specifications yields an average temperature offset of $6.15\,^{\circ }{\mathrm{F}}$ below temporal artery thermometer values when the device is held ${1}\,{\mathrm{ft}}$ away from the test subject’s forehead.

**Figure 6:**
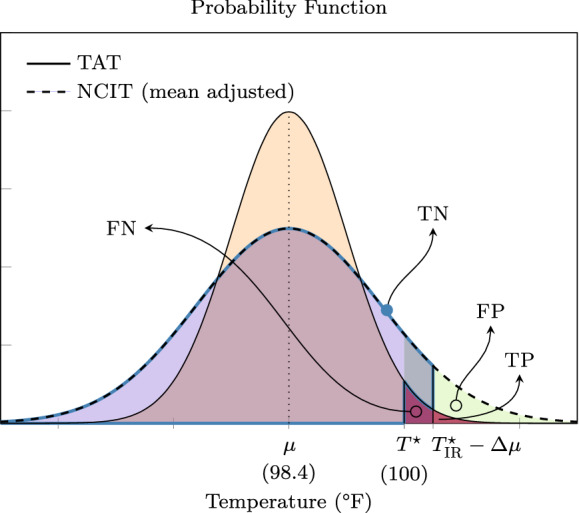
Overlay of probability distribution functions (PDF) for the reference measurement method, i.e. TAT, and the alternate or test measurement method, NCIT. Intersections of the PDFs define the FN, TN, FP, and TP groups given a referent fever screening temperature and a corresponding NCIT screening temperature. This plot illustrates the trade-off in sensitivity and specificity for temperature screening thresholds.

If the screening threshold is selected at $93.9\,^{\circ }{\mathrm{F}}$ in healthcare facilities, the Fluke 561 NCIT will provide a 60.5% sensitive and 80% specific test for the detection of temperature $\ge 100.0\,^{\circ }{\mathrm{F}}$ by temporal artery thermometry. Lowering the screening criterion to $91.1\,^{\circ }{\mathrm{F}}$ greatly increases sensitivity to 98.3% , but this is at the expense of an unacceptably high FPR of 82.9%, producing an inefficient test. This phenomena is concisely illustrated in Fig. 6.

Designing a reference standard for the measurement of temperature elevation in individuals being screened at healthcare facilities during COVID-19 using noncontact infrared thermometry to maintain spatial separation remains a value judgement.

At our institution, it is reasonable to propose a reference standard set to $93.5\,^{\circ }{\mathrm{F}}$ (Fig. 5). This would yield moderate test sensitivity and specificity of $70.9\%$, acknowledging a non-negligible FPR and FNR of $29.1\%$. Thus, the use of multi-step screening tools including issuance of symptom questionnaires and confirmatory TAT testing could further stratify individuals who screen positive by NCIT criteria.

That said, appropriate education and training of relevant staff, control of local conditions and environmental factors to reduce measurement error, and the acquisition of an NCIT with improved accuracy and a higher resolution D:S, such as the Fluke 568 with a distance to spot size ratio of 50:1 (see Table 1), may yield improved specificity while also maintaining a 6 ft distance between screening personnel and target individuals.

Individual facility resources and staff available for screening, population risk characteristics, epidemiological factors, and personnel throughput are factors that should be considered when selecting a screening criteria.

### Limitations

The following limitations were identified in our study:

(i) Because data collection was performed in the background of usual clinical activity, researchers did not adjust heating, ventilation, or air conditioning properties for each testing area per hospital ward.
(ii) Incident angle of measurement varied due to height difference between the researcher and participants.
(iii) Lack of blinding of researchers during temperature measurement and data collection.
(iv) Sample population contained no febrile observations, the majority of participants were female, and the sample was drawn from healthcare providers and therefore not a random distribution of the general population.

### Future work

A second-phase investigation is currently underway using the Fluke 568 infrared device (see Table 1), which has a D:S ratio of 50:1. Theoretically speaking, the main results of this paper extend to the Fluke 568 at $6\,{\mathrm{ft}}$ since the spot-size is comparable to the Fluke 561 at $1\,{\mathrm{ft}}$, and both devices have similar instrument accuracy. Thus, the aim is to validate the main results of this paper for temperature screening at further distances that comply with WHO and CDC separation guidelines (1 m and $6\,{\mathrm{ft}}$, respectively).

## Conclusions

Measuring skin surface temperature in mass public screening applications is an imperfect method for detecting elevated body temperature in individuals potentially infected with coronavirus. Doing so with an NCIT, in an effort to maximize social distancing and minimize risks of exposure to screening staff and healthcare personnel, introduces the potential for additional measurement error. However, a moderately sensitive screening test in the setting of high-risk pandemic virus is possible with NCIT. This requires proper device selection to match the intended application, i.e. specifying a D:S requirement that ensures a sufficient target spot size for the measurement distance, and determination of the device-specific temperature offset compared to an institution’s reference standard.

Under this method, the authors strongly advocate for appropriate training of staff, clear instructions-for-use, robust device calibration, and stabilization of environmental and procedural factors to increase success and maximize diagnostic accuracy.

The incorporation of an initial fever screening and subsequent quarantine, PPE, and sanitation regimens for febrile patients may prove beneficial beyond the course of this pandemic. A growing body of clinical research points to a decline in a number of infections normally endemic in hospital settings since the emergence of COVID-19. Studies such as the analysis of Clostridium difficile infection in health care settings by Bentivegna et al.^25^ attribute a statistically significant reduction of this most common pathogen in health-care associated infections with a combination of such practices. A positive externality of NCIT screening for COVID-19 induced fever likely has been the identification and isolation of other ‘sentinel’ fever producing pathogens and reduction of secondary infections. Such screening and measures may prove invaluable post COVID-19.

With respect to further research, the authors recommend future studies be inclusive of febrile patients and welcome collaboration testing the conclusions of this work. Data for febrile patients was necessarily synthesized for this study, but the authors hold that sound statistical methodology and understanding of the given technology was applied in drawing the conclusions presented. The authors also recommend the inclusion and analysis of NCITs from additional manufacturers, infrared cameras, and nascent technologies.

With respect to NCITs, institutions must realize this is just one of several requisite components of an effective screening program. At their most effective, NCITs such as the Fluke 561, may simultaneously achieve sensitivity and specificity results of approximately 70%. Thus, when employed properly, such tools may correctly identify a majority of febrile individuals, but a non-trivial number of febrile and afebrile persons will be misclassified. It is therefore crucial to understand that temperature measurement through IR thermometry is simply a supplemental screening mechanism. It is not a substitute for accurate and reliable diagnostic tests. For this reason, confirmatory temperature measurement by reference thermometry should be used for those who are identified as positive by NCIT. Lastly, multi-step screening tools such as exposure risk and symptom questionnaires are essential and necessary components of an effective program.

## Preliminaries

This study and the analyses herein were conducted and reported in the imperial system of units to ensure consistency of reporting in conjunction with U.S. federal agencies, healthcare agencies, and public and private healthcare systems. As such, all temperature measurements were taken, recorded, and analyzed in degrees Fahrenheit $(^{\circ }{\mathrm{F}})$ and all distances in feet (ft) or inches (in). Thus, we refer the reader to the following conversion factors.

Conversion from $T\, ^{\circ }{\mathrm{F}}$ to $T\, ^{\circ }{\mathrm{C}}$ is achieved through ${T}\, ^{\circ }{\mathrm{C}} = \frac{5}{9}({T}\, ^{\circ }{\mathrm{F}} - 32)$. The conversion factor for ft to meter is approximately $0.3048\,\tfrac{{\mathrm{m}}}{{\mathrm{ft}}}$.

Notable values used throughout the paper: $91.1\,^{\circ }{\mathrm{F}} \ (32.8\,^{\circ }{\mathrm{C}})$, $93.9\,^{\circ }{\mathrm{F}} \ (34.4\, ^{\circ }{\mathrm{C}})$, $96.7\,^{\circ }{\mathrm{F}} \ (35.9\,^{\circ }{\mathrm{C}})$, $100.0\, ^{\circ }{\mathrm{F}} \ (37.8\,^{\circ }{\mathrm{C}})$, and $1\,{\mathrm{ft}} \ (0.3048\,{\mathrm{m}})$, $3\,{\mathrm{ft}} \ (0.9144\,{\mathrm{m}})$, $6\,{\mathrm{ft}} \ (1.8288\, {\mathrm{m}})$.
